# The Effects of 12-Week Dual-Task Physical–Cognitive Training on Gait, Balance, Lower Extremity Muscle Strength, and Cognition in Older Adult Women: A Randomized Study

**DOI:** 10.3390/ijerph20085498

**Published:** 2023-04-13

**Authors:** Marcelo de Maio Nascimento, Paula Andreatta Maduro, Pâmala Morais Bagano Rios, Lara dos Santos Nascimento, Carolina Nascimento Silva, Matthias Kliegel, Andreas Ihle

**Affiliations:** 1 Department of Physical Education, Federal University of Vale do São Francisco, Campus Petrolina 56304-917, Brazil; 2 University Hospital of the Federal University of Vale do São Francisco, Campus Petrolina 56304-917, Brazil; 3 Department of Psychology, Federal University of Vale do São Francisco, Campus Petrolina 56304-917, Brazil; 4 Department of Psychology, University of Geneva, 1205 Geneva, Switzerlandandreas.ihle@unige.ch (A.I.); 5 Center for the Interdisciplinary Study of Gerontology and Vulnerability, University of Geneva, 1205 Geneva, Switzerland; 6 Swiss National Centre of Competence in Research LIVES—Overcoming Vulnerability: Life Course Perspectives, 1015 Lausanne, Switzerland

**Keywords:** aging, older adult, postural control, gait, falls, dual task, vulnerability, verbal fluency

## Abstract

This study aims to investigate the effects of dual-task physical–cognitive the training on body balance (BB), gait performance (GP), lower limb muscle strength (LEMS), and cognitive performance (CP) in a group of cognitively normal older adult women (*n* = 44; 66.20 ± 4.05 years). Of these, 22 were randomly allocated to the dual-task training (DT) group, and 22 participated in the control group (CG). Assessments were performed at baseline, after 12 weeks of intervention, and at the end of 12 weeks of follow-up, using the following instruments: Timed Up & Go (TUG), Timed Up & Go manual (TUGm), Timed Up & Go cognitive (TUGc), Balance Test (TEC), sit-to-stand test (STS), and verbal fluency test (VF). After 12 weeks of DT training, participants showed a significant time × group interaction in all motor assessments (BB, GP, LEMS), as well as in three cognitive tests (VF-grouping, VF-exchange, VF-total). No time–group interaction effect was indicated for the VF-category test. At all evaluation times, CG members maintained constant physical and cognitive performance. We conclude that 12 weeks of physical–cognitive DT training was effective in promoting BB, GP, and LEMS, as well as CP in cognitively normal older adult women, with lasting effects up to 12 weeks after the intervention.

## 1. Introduction

Aging brings a series of biological, psychological, and functional changes [1], making the individual more vulnerable to a series of changes in their health status [2,3]. Thus, compared to young and adult individuals, older adults tend to have functional disabilities and limitations [4], making it difficult to perform their activities of daily living (ADLs) in an agile way [5]. Among the alterations that the natural aging process entails are gait performance (GP) [6,7] and body balance (BB) [8,9]. In advanced age, the adequate performance of both motor capacities is essential for the individual to independently perform ADLs free from accidents, including falls [8,10]. In this context, cognition plays an important role since motor functions (i.e., gait and balance) are regulated by neural pathways [11,12]. Moreover, deficits in the functioning of the nervous system can limit the recruitment of motor units [13], increasing the risk of falls. Muscle strength plays a strong role during aging; in the clinical area, its close relationship with cognitive performance is often assessed by handgrip strength, serving as a biomarker of cognitive impairment [14].

Cognitive changes caused by aging are characterized by a reduction in synaptic density and the dendritic arborization of cortical neurons [15], which leads to structural changes in the brain (i.e., decreased white matter volume). In turn, these modifications consequently reduce cognitive performance (CP). CP is a comprehensive term, and one of its components is the executive functions (EFs), defined as a set of integrated cognitive skills necessary for the control and regulation of common everyday actions, such as thoughts, emotions, and conflicts [16]. Low EF performance restricts the domain of tasks that require working memory, conceptual reasoning, attention, visual and spatial scanning, as well as verbal fluency skills [17,18]. In practice, the impairment of EFs impairs neuronal transmission, predisposing the older adult to lapses mainly in areas of the prefrontal cortex [19], reflecting negatively on the mechanisms responsible for GP [20,21] and BB [22]. It is worth mentioning that, even in cognitively healthy older adults, small structural and functional changes in the brain can affect the performance of EFs [18,23,24].

The relationship between motor tasks such as GP and BB with cognition is controlled by muscle modulation processes, known as muscle synergy [25]. Due to physiological aging, sensory and motor organs undergo gradual changes, which require adapted synergies from the body for proper functioning. For this reason, older adults have a more cautious motor control [26], which is a response to the barriers that both the environment and motor coordination itself impose on the brain [27]. Depending on the older adult individual, the task of walking at an adequate speed, simultaneously coordinating the reciprocal swing of the arms with the extension/stabilization of the pelvis and ankles, can be a great challenge [28,29]. In general, changes in CP make older adults more vulnerable to managing complex activities [30], especially those involving dual-tasking (DT) [31]. DT is defined as the ability to simultaneously perform two or more concurrent tasks [32]. Solving different tasks at the same time requires a preserved CP to process perceptual, motor, and cognitive mechanisms in an integrated way [33]. Therefore, a low CP may increase the risk of delayed motor control responsible for GP [34,35], as well as partial inhibition of the functioning of the visual, somatosensory, and vestibular systems, responsible for BB regulation [36].

In the clinical area, the use of simultaneous tasks that compete for limited resources (i.e., dual-task interference) is presented as a strategy to identify cognitive deficits [37,38]. In turn, physical–cognitive DT training proved to be a much more effective measure than simple task training to improve GP and BB in the older adult population [39,40]. Therefore, physical–cognitive DT training was also found to be effective in preventing falls [41,42]. When it comes to falls, it is worth noting that aging also changes the strength level of the lower extremity musculature (LEMS). This degenerative process is defined as sarcopenia, which consists of the natural loss of muscle mass [43]. Changes occur at cellular, neural, metabolic, and hormonal levels, causing a loss of type II muscle fibers, which in turn generates weakness and low muscle power [44]. Proper LEMS performance is a prerequisite for maintaining an upright posture [45]. Furthermore, adequate levels of LEMS are essential for the execution of a fast and safe gait [46,47].

In general, a low CP and GP [48,49], BB [50], and LEMS [46] are factors for increased risk of falling. In the older adult population, falls are responsible for injuries, fractures, and days of hospitalization [51], with possible future restrictions of mobility [52], which may hinder the individual’s independence. Moreover, falls are one of the leading causes of death among older adults [53]. The literature also highlights that falls are more prevalent among women [52,54]. Although review and meta-analysis studies have highlighted the benefits of the DT methodology as a useful strategy to activate brain structures [42,55] and concomitantly GP and BB [56,57], as well as LEMS [44], there are still gaps, in addition to the fact that definitive conclusions were limited by the lack of studies that have investigated the reciprocal effects between all the variables [58,59]. Thus, to date, no studies have specifically examined the effects of physical–cognitive DT training on GP, BB, LEMS, and cognitive vulnerability in a population of cognitively normal older adult women.

The purpose of this study was to examine the effect of a 12-week physical–cognitive DT training program on GP, BB, LEMS, and cognition in a group of cognitively normal older adult women. We hypothesized that, after 12 weeks of intervention, members of the physical–cognitive DT training would indicate better performance in all tests. In turn, comparatively, members of the DT group would present better results in the evaluations after 12 weeks of follow-up than members of the control group. We justify the single inclusion of older adult women in the study because both in Brazil, as well as in the city where this investigation was carried out, there is a multifaceted phenomenon called the feminization of aging [60]. Thus, in Brazilian cities, the number of women (age ≥ 60 years) tends to be higher than that of men [61]. Another peculiarity is that it has been proven that there is a higher risk of falling among women [62].

## 2. Materials and Methods

This is a randomized experimental study with a double-blind design. Those responsible for the evaluations in all phases of the study were blinded. After assessments of all participants at baseline (T1), study members were randomly assigned (1:1) into a dual-task (DT) or control (CG) group. Single-sequence randomization was generated by a web-based search randomizer (www.randomizer.org (accessed on 30 July 2018), conducted by an investigator blinded to both study objectives and recruitment. The effectiveness of a 12-week DT training intervention was determined by post-intervention (T2) assessments. Finally, 12 weeks after the end of the intervention, follow-up assessments (T3) were performed to detect the potential long-term effects of DT training on the variables of interest. The study was carried out between October 2018 and March 2019. Members of the DT and CG group were instructed to perform only their daily activities during the follow-up period.

### 2.1. Sample Size

Sample size and power calculations were performed with G* Power3, based on a previous study. Thus, a priori repeated measure ANOVA suggested that a total sample size of 44 was needed to reach 0.80% power to detect the interaction effect size of 0.20 at the 0.05 level of significance. Considering possible sample loss, 50 individuals were evaluated and allocated into the two groups in the proportion of 1:1.

### 2.2. Participants and Eligibility

Participants were recruited through dissemination in WhatsApp, Facebook, radio, and older adult groups in the city. Initially, the intention was to include individuals of both sexes, however, 98% of those interested were women. Thus, the investigation was composed exclusively of older women. It is worth noting that in Brazil, the location of this investigation, a multifaceted phenomenon called the feminization of aging has been observed [60]. Thus, the number of older adult women (≥60 years) is greater than that of men [63]. Consequently, Brazilian women also present a higher risk of falling than men [62], which strengthens the execution of this investigation. Thus, the inclusion criteria adopted were: (1) sex: women; (2) age: 60–79 years; (3) Berg Balance Scale: score >52 (out of 56) [64]; (4) ability to walk without aids for 10 min at a minimum speed of 1 m/s; and (5) Mini-Mental State Examination scores: score of 20 points (out of 30) for participants were illiterate, 24 points for those with one to four years of education, and 26.5 points for older adults with five or more years of education [65]. Exclusion criteria were: cardiovascular, musculoskeletal, or severe neurological diseases (such as Parkinson’s and multiple sclerosis); significant hearing or visual impairment; and participation in a regular strength, balance, or dual-task training program 6 months before the start of the study. All participants were informed about the objectives and risks of the study and signed the consent form before any evaluation was applied, according to the Declaration of Helsinki. Participants received no financial compensation.

### 2.3. Intervention

#### 2.3.1. Dual-Task Training

DT physical–cognitive training was structured based on the principle of variable priority, when the focus of attention is divided between two or more tasks [33,56]. Thus, motor tasks (gait and balance) were associated with cognitive strategies (internal interference factors). Our training protocol was presented in a previous study [66]. Table 1 presents the set of activities carried out in the physical–cognitive dual-task training. DT group underwent activities twice a week (60 min/section). The training protocol was performed in a fitness room (20 × 20 m). The activities were accompanied by a team of trained professionals in the area of Physical Education and Physiotherapy. A training section had three phases: (1) warm-up (10 min): supervised walking exercises on a flat surface, general joint mobilization, and stretching exercises; (2) DT training (40 min): performance of exercises (i.e., balance and gait) to simulate daily actions with risk of falling, associated with the simulation of actions that prioritized the exchange and transfer of tasks; and (3) return to calm (10 min): relaxation and breathing exercises were performed with participants lying on the floor. 

The training load was implemented by the progression of tasks, more specifically, through the variation in motor exercises and increased difficulty of cognitive tasks. This strategy was modified every two weeks, more specifically, after four training sessions. Thus, we seek to intensify neuroplasticity, reducing the interference of competitive demands at the neural level, and favoring the exercise of allocation simultaneously from two or more tasks. Table 2 describes the set of strategies used to increase the load of gait and balance exercises. Moreover, we include two strategies to strengthen the social component, as well as the adherence of participants to training: (1) the offer of activities through a DT circuit formed by five stations, and (2) the distribution of participants in groups of 5–6 individuals. Next, the groups performed the tasks for 8 min (march, balance) in each of the five seasons. We emphasize that the procedures for diversification and combination of tasks at the stations (Table 1), as well as the progression of the task load (Table 2), were also intended to differentiate the set of DT training exercises from the tasks required in the motor tests.

#### 2.3.2. Education Control Group

The CG activities consisted of a set of thematic workshops developed by the interdisciplinary team of the University of Third Age. The activities were under the responsibility of professionals from different areas (Physical Education, Medicine, Pharmacy, Nursing, Physiotherapy, History, Literature, English, Politics, Sociology, Informatics, and Arts). The methodology used was guided by the pedagogical principles of permanent education specific to the adult older population [67]. These activities valued the meeting with the exchange of information. The activities took place twice a week (60 min sessions). During all stages of the study, the CG received no physical or cognitive training and was instructed to continue its usual activities. Throughout the study (24 weeks), members of the CG were not notified of any adverse event, including falls.

### 2.4. Control of Procedures and Adherence

Members of both groups attended at least 75% of training sessions [68]. The DT group indicated 94% adherence to the training over 12 weeks, and participants in the CG group had 92% participation in the thematic workshops. Once a month, participants in both groups were asked about their satisfaction with the activities. The strategies used to maintain the adherence of all participants to the study during the follow-up period were as follows: (1) weekly contact via WhatsApp or by phone and (2) sending three booklets with cultural and health information. All participants were instructed not to perform any type of physical or cognitive training during the 24 weeks of the study; therefore, they were instructed to just continue with their daily activities. Throughout the entire study, no adverse events, including falls, were accounted for.

### 2.5. Outcome Measures

Before randomization, participants answered a standardized questionnaire to collect sociodemographic data (e.g., age, education), history of falls, medications, and comorbidities. Secondly, anthropometric data (weight, height) were collected, and the Body Mass Index (BMI) was determined. Finally, we used the Mini-Mental State Examination (MMSE) [65], considering the following scores: 20 points for participants who were illiterate; 25 points for individuals with an education between 1 and 4 years; 26.5 points for those aged 5 to 8 years; 28 points for 9 to 11 years of education; and 29 points for those with more than 11 years of education. The data for this investigation were collected by duly trained team examiners with experience in applying motor and cognitive tests.

#### 2.5.1. Primary Outcomes

The instruments used in this study are part of the list of tests commonly applied to assess mobility [69], examine the sensory functions of static and dynamic balance [70], predict the risk of falls due to muscle weakness [Power et al., 2014], and assess cognitive functions [71]. The primary outcome was defined by spatiotemporal variables, conventional TUG (TUG), manual TUG (TUGm), and cognitive TUG (TUGc); the assessment of static and dynamic BB was obtained by the postural balance test (TEC) and the assessment of the underlying cognitive structure of phonemic fluency and semantic memory functions was obtained through the verbal fluency test (VF). Regarding the gait assessment, in all three tests, participants were instructed to get up from a chair (seat height 40–50 cm), walk 3 m in front of them, turn around in a cone, walk back to the chair, and sit down. The time for each test was recorded using a 0.01 s precision timer. The measurement started when the participant’s back left the chair and ended when the participant returned to sit with their back touching the chair. A practical test was performed for each TUGm test, and the order of evaluation of the three tests was randomized. The assumed final score was the average time between two attempts. The gait assessment in dual-task conditions includes the TUGm and TUGc tests [72].

In the TUGm, participants were asked to travel the 3 m-round trip carrying a paper tray (25 cm in diameter) with a dominant hand. On the tray, there was a glass (6.9 cm in diameter and 8.4 cm high) containing 200 mL of water (1 cm below the edge). In the TUGc, the patients were instructed to repeat the days of the week in reverse order, starting on Sunday. 

The performance of static and dynamic balance was evaluated by the Gleich Gewichsttest, validated by Wydra [73] with the independent German population (*n* = 306), presenting a test–retest reliability of 0.78 (α Cronbach = 0.92). The test was presented to the Portuguese-speaking community as the TEC-Body Balance Test [74]. The TEC test has 14 tasks—7 assess static balance and 7 assess dynamic balance. Six tasks examine the performance of the sensory regulation system with an emphasis on vision and eight assess the performance of the vestibular and somatosensory systems. The test score is dichotomous, so the individual receives one (1) point for each task achieved and zero (0) for those not completed. The risk of falling for women (60–79 years) is determined by the performance ≤ 4 tasks. 

The VF test examined the phonemic fluency and semantic memory system’s ability to strategize, organize, process, and retrieve information stored in long-term memory [75]. Participants were asked to recall, in one minute, the largest number of known animal names. The performance analysis process was as follows: (1) VF-total: the total score of named animals (60 s); (2) VF-category: the number of categories formed, that is to say, the evocation of animal names representing a particular semantic subcategory; (3) VF-grouping: the number of groupings performed; and (4) VF-exchange: the number of exchanges performed. Other results related to the executive functions arising from the training protocol of the present study were previously presented [66].

#### 2.5.2. Secondary Outcomes

LEMS was performed using the sit-to-stand (STS) test [76]. During the performance of the STS, participants were asked to be barefoot to eliminate possible footwear effects. Thus, the participants were instructed to sit in a chair (12-inch high) with their arms placed at their sides. Participants were asked, after a signal, to stand up as quickly as possible without any help from the arms, and then return to a fully seated position, repeating this action as many times as possible for 30 s. The evaluation was performed three times: the mean scores were used for data analysis. In addition to providing information on lower limb strength, the STS informs the subject’s ability to transfer their center of gravity (COG) from a sitting position to a standing position.

### 2.6. Covariates

Considering possible confounding factors, the analyses were controlled by a set of variables recognized as a potential for falls among the older adult population, namely: sex and age [77,78], and years of education [79,80]. For the analysis, this confounding factor was inserted as a continuous variable. Other covariates were the number of falls in the last 12 months [81], the number of different types of medications consumed daily [82,83], and comorbidities [84,85]. All this information was obtained through face-to-face interviews.

### 2.7. Statistical Analysis

Normality was tested with the Shapiro–Wilk test. Categorical variables (medications, education, and comorbidities) were presented by several cases, and the frequency was analyzed using the chi-square test. Considering that all scalar variables (age, BMI, falls, MMSE, BB, GP, VF) presented a normal distribution, they were acquired through means and standard deviations and were analyzed using the t-test for independent samples, while categorical data were analyzed using the chi-square test. The estimation of between- and within-group differences at baseline, post-training, and 12-week follow-up was performed by three-way repeated measures ANOVA. For this purpose, we considered the intervention DT versus CG as a factor between groups, and the measurement of time as a factor within the group (2 × 3 comparison). Data processing took place after checking all assumptions (homoscedasticity and normal distribution). 

Using partial eta-square, intergroup effect sizes were calculated according to the following categorization: small (*η_p_*^2^ = 0.01), medium (*η_p_*^2^ = 0.06), and large (*η_p_*^2^ = 0.14) [86]. Therefore, we included ANOVA (Bonferroni post hoc) analyses to control for the possible locus of significant difference between two assessment times. These analyses were adjusted for confounding factors (i.e., age, years of education, falls, medications, and comorbidities). The significance level adopted was *p* < 0.05. Data analysis was performed using Statistical Package for the Social Sciences (SPSS) version 24.0.

## 3. Results

**Figure 1:**
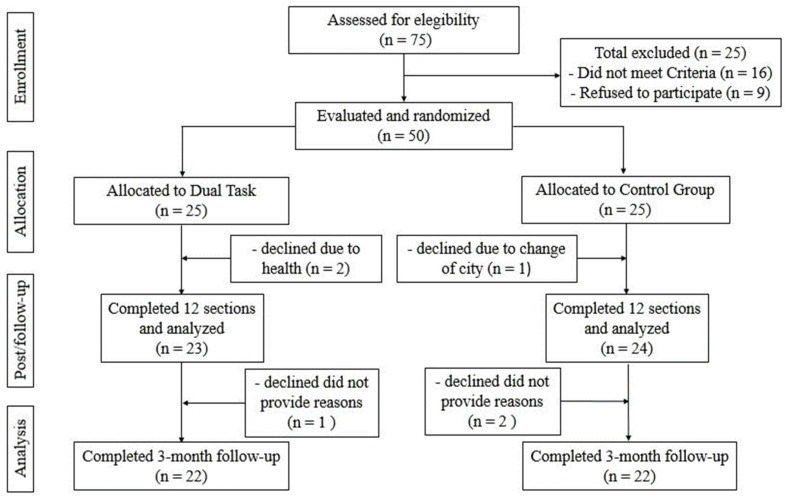
Study design and flow of participants.

Seventy-five people were selected for the study (Figure 1). Of these, fifty met the inclusion criteria and were randomized. Finally, 25 people were allocated to each of the 2 groups. The analyses presented refer to 44 subjects (DT = 22, GC = 22) who completed the 24 weeks of the study.

### 3.1. Sample Characteristic

Forty-four women participated in the study (66.20 ± 4.05 years). No significant difference between the groups was found in the baseline assessments (*p* > 0.05) (Table 3). There was an average level of education between 3 and 4 years. According to the total MMSE score, preserved cognitive performance was observed in both groups (25.30 ± 2.68 points). The daily consumption of medication varied between 1 and 2 types, and the history of falls in the last 12 months was low (0.16 ± 0.37). Among the comorbidities, the highest prevalence was verified for rheumatism (72.7%), diabetes mellitus, visual acuity, hearing, osteoporosis, and hypertension (50.0%). All results at baseline showed a significance level of *p* > 0.050.

### 3.2. Descriptive Statistics

#### 3.2.1. Gait, Body Balance, and Lower Extremity Muscle Strength

**Figure 2:**
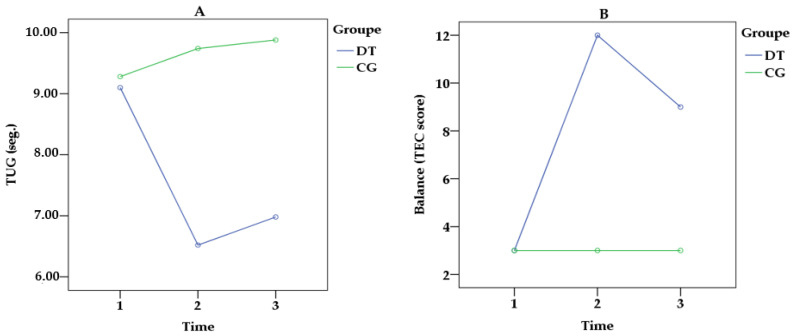
Results of the time × group interaction analysis, according to the groups. (**A**) Single gait performance by the TUG test; (**B**) performance of static and dynamic balance by the TEC test; seg. = seconds.

The comparative results obtained by both groups in the motor assessments at baseline, post-tests, and follow-up are presented in Table 3. Conventional TUG showed significant time–effect differences [F(2.96) = 9.043, *p* = 0.006, *η_p_*^2^ = 0.698] and a significant group × time interaction [(2.96) = 10.470, *p* < 0.001, *η_p_*^2^ = 0.768] (see Figure 2A). According to the post hoc analysis adjusted for possible confounders (i.e., age, years of education, falls, medications, and comorbidities), members of the DT group showed significant results between T1 and T2 (*p* < 0.001) and from T2 to T3 (*p* < 0.001). Regarding gait in the dual-task condition, the TUGm indicated significant time–effect differences [F(2.96) = 10.638, *p* = 0.008, *η_p_*^2^ = 0.652] and a significant group × time interaction [F(2.96) = 8.321, *p* < 0.001, *η_p_*^2^ = 0.745]. Post hoc analysis adjusted for confounders (i.e., age, years of education, falls, medications, and comorbidities) indicated significant results between T1 and T2 (*p* < 0.001), and from T2 to T3 (*p* < 0.001). TUGc pointed significant time–effect differences [F(2.96) = 8.695, *p* = 0.021, *η_p_*^2^ = 0.622] and a significant group × time interaction [F(2.96) = 9.452, *p* = 0.014, *η_p_*^2^ = 0.738. Therefore, the post hoc analysis adjusted for confounders (i.e., age, years of education, falls, medications, and comorbidities) showed significant results from T1 to T2 (*p* < 0.001) and from T2 to T3 (*p* < 0.001). The largest effect size generated by dual-task physical–cognitive training was indicated in the BB test. Our analysis showed a significant time–effect difference [F(2.96) = 10.143, *p* < 0.001, *η_p_*^2^ = 0.939] and group × time interaction [F(2.96) = 11.125, *p* < 0.001, *η_p_*^2^ = 0.989] (see Figure 2B). Specifically, in the DT group, the post hoc analysis adjusted for confounders (i.e., age, years of education, falls, medications, and comorbidities) pointed toward significant results from T1 to T2 (*p* < 0.001) and from T2 to T3 (*p* = 0.032). Finally, for LEMS, we found a significant effect on time [F(2.96) = 11.024, *p* = 0.000, *η_p_*^2^ = 0.892] and a significant group × time interaction [F(2.96) = 12.108, *p* = 0.000, *η_p_*^2^ = 0.934]. According to the post hoc analysis adjusted for confounders (i.e., age, years of education, falls, medications, and comorbidities), members of the DT group showed significant results from T1 to T2 (*p* < 0.001), and a significant result from T2 to T3 (*p* < 0.001). Meanwhile, CG members maintained their performance unchanged over 24 weeks (Table 4).

#### 3.2.2. Cognition

Concerning the result of the VF-total, there were significant time–effect differences [F(2.96) = 6.301, *p* = 0.018, *η_p_*^2^ = 0.334] and significant group × time interactions [F(2.96) = 9.754, *p* = 0.000, *η_p_*^2^ = 0.388] (see Table 3 for an overview). According to post hoc analysis adjusted for confounders (i.e., age, years of education, falls, medications, and comorbidities), members of the DT indicated significant results from T1 to T2 (*p* ≤ 0.001), and from T2 to T3 (*p* < 0.001). The VF-category did not show any significant time–effect differences on time [F(2.96) = 4.622, *p* = 0.079, *η_p_*^2^ = 0.282] and in the analysis of the group × time interaction [F(2.96) = 4.109, *p* = 0.145, *η_p_*^2^ = 0.298]. VF-grouping showed significant time–effect differences [F(2.96) = 6.482, *p* = 0.032, *η_p_*^2^ = 0.196] and group × time interactions [F(2.96) = 6.278, *p* = 0.038, *η_p_*^2^ = 0.214]. Post hoc analysis adjusted for confounding factors (i.e., age, years of education, falls, medications, and comorbidities) indicated significant results from T1 to T2 (*p* = 0.024) and from T2 to T3 (*p* = 0.018). Finally, the VF-exchange pointed toward significant time–effect differences [F(2.96) = 8.781, *p* = 0.002, *η_p_*^2^ = 0.189] and a significant group × time interaction [F(2.96) = 8.253, *p* = 0.012, *η_p_*^2^ = 0.247]. Post hoc analysis adjusted for confounding factors (i.e., age, years of education, falls, medications, and comorbidities) showed significant results from T1 to T2 (*p* ≤ 0.001) and from T2 to T3 (*p* ≤ 0.001) (Table 3).

## 4. Discussion

This study investigated the effects of a 12-week dual-task physical–cognitive training on gait performance, balance, lower extremity muscle strength, and cognitive performance in a group of cognitively normal older adult women compared to a control group, who participated only in educational activities. Our two assumptions were confirmed. First, we found that DT training was able to generate significant time–group interaction effects after the 12-week intervention on the performance of BB, GP, LEMS, and EFs, except for the VF-category. Second, members of the DT training also reported better results on these same variables at the end of the 12-week follow-up. Thus, our findings were in line with previous investigations that showed concurrent physical–cognitive training as being capable for neutralizing age-related deterioration in physical and cognitive functions [87,88]. 

A strength of our study was the training protocol. When it comes to DT training, the type of exercise applied is decisive for the creation of internal interference factors [88]. A systematic review study highlighted that the effectiveness of a DT training program depends on the exercise load [42]. Therefore, factors such as task intensity and duration are critical, which include increasing levels of difficulty, as well as the proper planning of prioritizing task specificity. In this sense, our results showed that the set of motor and cognitive tasks as well as the progression strategies were able to stimulate the sharing of complex neural networks in different regions of the brain [89]. Therefore, the training program was able to reduce the interference of competitive demands generated by motor and cognitive tasks, increasing the ability of DT group members to allocate attention between two or more tasks performed simultaneously [90]. Another positive point of the applied training was the reciprocal benefits for the GP [91], BB control [92], LEMS [44], as well as the efficiency of the functions cognitive [42]. 

It is noteworthy that although the performance of the CG members on all outcome variables (T2 and T3 assessments) was lower than the results of the DT group, except for BB, their performance levels were adequate for an age group between 60 and 79 years. In our study, while DT group members indicated an improvement in gait performance in a single task, motor dual task, and cognitive dual task, CG participants maintained gait speed in the three different conditions unchanged. Walking is a complex task that requires a mutual work of the sensorial and cognitive systems [93]. Changes in speed (slowness) during the simultaneous execution of two or more different tasks (motor and cognitive) reflect an increase in the cost of the dual task [94,95]. Thus, a walking speed below 1.0 m/s is considered abnormal [95]. 

In this study, the benefits of DT training became more evident after the follow-up period, when lasting effects (retention gains: Δ = T3 − T1) were revealed. Thus, members of the DT group indicated in the gait condition a single task (gain of 23.3%), motor dual task (gain of 20.9%), and cognitive dual task (gain of 12.2%). Similar results of DT training in GP among older adults have also been reported in previous experimental studies [58]. In a randomized controlled trial conducted with cognitively normal older adults (*n* = 37, 60–75 years) that compared the effects of six weeks of single-task and dual-task training on gait and cognitive performance, the DT training group indicated better GP (i.e., speed, cadence, step length) in single-gait conditions and with dual-task interference [91]. 

In the present study, the greatest power effect revealed by DT training was observed in the BB test. The improvement in balance control can be attributed to the set of tasks experienced by the older adults in the DT group, which improved the functioning of the sensory system organs responsible for regulating postural control (i.e., visual, auditory, and somatosensory systems). Thus, over 24 weeks, while the CG members maintained their balance performance unchanged, those who participated in the DT training increased the efficiency of this motor skill by up to 300.0% from T1 to T2, and were able to retain the gains (Δ = T3 − T1) by up to 200% after follow-up. Our findings are in line with previous studies [96,97], showing that training based on DT principles was able to significantly benefit the postural control of cognitively healthy older adults. 

The improvement in the BB performance was in agreement with a previous review study [98], since the applied DT protocol benefited neurocognitive functions responsible for mechanisms underlying postural control. Among them, sensory organs such as muscle and joint proprioceptors (somatosensory system) were responsible for capturing postural information before sending the information to the brain [99,100], including graviceptors located in the trunk [101]. In turn, the vestibular system, fundamental for trunk flexion and extension actions, as well as sensitive to angular and linear acceleration of the head position (gravity), was also benefited [102]. Another stimulated sensorial organ was the optic nerve, which is an auxiliary in postural stability actions and a determinant to identify obstacles in the planning of the set of trajectories during gait [103].

Another positive effect of DT training was for LEMS. With aging, there is a gradual loss of related muscle strength that can compromise performance in ADLs, even reducing perceived quality of life [104]. Extremely low levels of muscle strength are increasing the frailty of the older adult population [105]. For this reason, their participation in resisted physical training is advised [44]. Thus, individuals may become used to transferring physical gains from training to ADL [106]. In a study that evaluated the effects of gait self-efficacy and lower limb physical function on DT performance (*n* = 195, 60–79 years), greater self-efficacy for LEMS was found in the DT condition [107]. Furthermore, performance on DT tasks was predictive of safe street crossing. In an observational study carried out with 1705 community-dwelling older adults (70 years old), an association was found between low quadriceps strength and self-reported functional limitation [108]. Thus, our results showed that while CG members maintained constant LEMS performance for 24 weeks, those who integrated DT training increased performance by up to 81.1% from T1 to T2 and were able to retain strength gains (Δ = T3 − T1) by up to 45.4% after follow-up. The findings are significant, confirming the benefits of DT training as a possible strategy to counteract strength losses resulting from sarcopenia and/or physical inactivity among older adults [43].

Regarding cognition—more specifically, a subcomponent of executive functions, which is verbal fluency—we found that, except for the VF category, while the cognitive performance of the CG members remained unchanged over 24 weeks, the members of the DT training indicated improvement in the performance of phonemic fluency and semantic memory functions. The effective gain can be verified after follow-up through retention effects (Δ = T3 − T1) of up to 26.6%, 176.7%, and 116.6%, respectively, in the categories VF-total, VF-category, and VF-grouping. The literature has highlighted the benefits of physical exercise under the principles of DT to increase the cognitive reserve of the older adult population [94,109]. Through gait and balance tasks associated with cognitive strategies, it was possible to create challenges capable of promoting the neural plasticity of the members of the DT group. 

It is worth noting that, based on the MMSE test, all of our study population had a preserved cognitive state. Therefore, they experienced natural aging [110]. However, even among cognitively healthy older adults, having a preserved capacity for building clusters and semantic exchanges is fundamental, since physiological aging generates different degrees of deficits in the frontal neural pathways, of which are responsible for the performance of working memory, mental flexibility, deviation of attention, visual processing speed, and exploration [111]. Thus, based on the results of the three VF subtests and on the total VF score, it was possible to verify that the applied training protocol was effective for the performance of verbal memory (temporal lobe), capacity for procedural change (frontal lobe), and cognitive flexibility [112]. All of these functions are crucial to the effectiveness of cognitive flexibility [93].

The present study has some limitations. First, findings from the present study can only be generalized to a specific group of older adult women, and only for those who meet the same selection criteria adopted in this investigation. Second, considering that the cognitive and motor tests were applied three times over a period of six months, it is possible that some participants memorized the tasks, generating bias in the results. Third, neuroimaging techniques were not added to further monitor the neural changes caused by DT training. Thus, it must be considered that it is possible that the clinical instruments used did not offer an in-depth analysis of the underlying brain mechanisms that benefited from DT training. Fourth, the levels of complexity, specificity, and prioritization of the motor and cognitive task limit the comparability with previous studies. Thus, the effect sizes generated by our physical–cognitive DT training protocol may vary considerably from those presented by other studies. Fifth, the use of only one cognitive test is a limitation of this investigation. Therefore, it is suggested that future studies, in addition to the verbal fluency test, add other instruments to verify the effects of physical–cognitive DT training on executive functions. The measure can broaden the understanding of the underlying mechanisms of neural plasticity generated by this type of training. Finally, it is encouraged to carry out a longitudinal follow-up of the evaluated population in order to state more precisely the effects of DT training and natural aging on GP, BB, LEMS, and EFs.

## 5. Conclusions

The findings provided evidence for the effectiveness of 12 weeks of dual-task physical–cognitive training in counteracting age-related changes in gait performance (single- and dual-task conditions), also improving static and dynamic balance performance, and increasing lower limb strength. A strong point of our study was the non-requirement of expensive equipment in the training protocol, which also ensured the participants’ adherence over the 12 weeks. In turn, admitting that physiological aging is responsible for changes in cognitive functions, enhancing the loss of autonomy and vulnerability of the individual, DT training proved to be capable of inducing neural plasticity (i.e., search and retrieval of data, ability for organization, self-regulation, and operational memory). Our results can serve as a reference for new investigations focused on the prevention of falls in cognitively normal older adult women.

## Figures and Tables

**Table 1:** Description of training components and task specificity.

Focus	Training Tasks
Gait	(a) walking with short and wide steps, on the heels and tiptoes, on the back
Static balance	(a) biped, semi-tandem, tandem, single leg stance, weight on feet (hip and ankle postural stabilization strategies); (b) careful inclusion of switching between open and closed eyes.
Dynamic balance	(a) walking in different directions on a soft, unstable, or reduced surface (hip and ankle postural stabilization strategies); (b) inclusion of arm movements outside the center of pressure.
Cognitive task	(a) calculations and countdown (100, 97, 94, 91, 89, …); (b) verbal fluency (name fruits, people, or cities starting with different letters of the alphabet); (c) memorization (memorizing a sequence of 3–5 different words and after reproducing); (d) visual tasks and word spelling (i.e., reaction time: react as quickly as possible), talking with colleagues.

**Table 2:** Strategies to increase DT training load during gait and balance tasks.

Focus	Strategies for Progression
Gait	(a) slow or fast, with short or wide steps, on the heels or on the tip of the feet, forward, backwards, low or high level, diagonals, overtake obstacles; (b) perform curves and/or turns (180°, 360°); (c) combine with the manipulation of objects, or take objects on the floor; (d) sensory input: impaired vision, enhancement of somatosensory integration.
Static balance	(a) variation in the demand of hip and/or ankle strategies; (b) variation in surfaces: soft/hard, stable/unstable, wide/reduced.
Dynamic balance	(a) walk combined with arm movements outside the pressure center (COP); (b) backward gait; (c) surface change: soft/hard, stable/unstable, wide/reduced.
Cognitive task	(a) progressive increase in the difficulty of counting/memorization tasks; (b) order/sequence of numbers/words; (c) variation in response time; (d) alternation of task length; (e) Stroop tasks (i.e., an alternating combination of incongruent and congruent tasks).

**Table 3:** Main characteristics of participants in the baseline.

Variable	Dual-Task (*n* = 22)	Control Group (*n* = 22)	*p*-Value
Age (years)	66.14 ± 4.15	66.27 ± 4.04	0.913
BMI (kg/m^2^) (n)	27.68 ± 3.93	28.18 ± 4.67	0.703
Falls (12 months) (n)	0.27 ± 0.19	0.185 ± 0.21	0.132
Medication n (%)			0.161
1–4 types	20 (90.9%)	19 (86.3%)	
>4 types	2 (9.0%)	3 (13.6%)	
Education n (%)			0.574
1–4 years	3 (13.6)	4 (18.1)	
≥5 years	19 (86.3)	18 (81.8)	
MMSE (n)	25.27 ± 1.38	25.32 ± 3.57	0.688
Comorbidities n (%)			
Diabetes Mellitus Yes (f)	4 (18.1)	18 (81.8)	0.545
Hypertension Yes (f)	9 (40.9)	13 (59.0)	0.680
Visual acuity Yes (f)	20 (90.9)	2 (9.0)	0.761
Hearing			0.550
Yes (f)	11 (50.0)	12 (54.5)	
Labyrinthitis Yes (f)	4 (18.1)	2 (9.0)	0.079
Osteoporosis Yes (f)	14 (63.6)	8 (36.3)	0.294
Rheumatism			0.488
Yes (f)	6 (27.2)	16 (72.7)	

Note: Data are expressed as mean (M) ± standard deviation (SD) or frequency (f); kg = Kilograms; cm = centimeters; MMSE = Mini-Mental State Examination; BMI = Body Mass Index; kg/m^2^ = kilograms/square meter; *p* < 0.005 = Mann–Whitney U test.

**Table 4:** Results between the dual-task and control groups for gait, balance, lower extremity muscle strength, and cognitive performance.

Variable	Dual-Task			Control Group			Time			Time * Group		
	(Baseline)	(12 Weeks)	(24 Weeks)	(Baseline)	(12 Weeks)	(24 Weeks)	F	*p*	*η_p_* ^2^	F	*p*	*η_p_* ^2^
	*n* = 22	*n* = 22	*n* = 22	*n* = 22	*n* = 22	*n* = 22						
Gait												
TUG (s)	9.10 ± 1.88	6.52 ± 0.79	6.98 ± 1.17	9.28 ± 1.59	9.74 ± 1.59	9.88 ± 1.31	9.043	0.006	0.698	10.470	<0.001	0.768
TUGm (s)	9.68 ± 2.14	6.74 ± 0.89	7.66 ± 1.32	9.55 ± 1.46	9.82 ± 1.26	9.94 ± 1.21	10.638	0.008	0.652	8.321	<0.001	0.745
TUGc (s)	11.14 ± 2.43	9.31 ± 0.86	9.78 ± 1.41	11.18 ± 2.25	11.25 ± 1.53	11.10 ± 1.78	8.695	0.021	0.622	9.452	0.014	0.738
Balance												
TEC	3.00 ± 2.5	12.00 ± 2.7	9.00 ± 3.1	3.00 ± 2.0	3.00 ± 2.4	3.00 ± 2.0	10.143	<0.001	0.939	11.125	<0.001	0.989
Muscle strength												
LEMS	11 ± 4.2	20 ± 3.1	16 ± 4.3	11 ± 3.4	11 ± 3.5	11 ± 3.2	11.024	<0.001	0.892	12.108	<0.001	0.934
Cognition												
VF-Total (n)	15.00 ± 3.7 ^b,c^	20.00 ± 4.0	19.00 ± 3.1	15.00 ± 2.9	16.00 ± 3.2	15.00 ± 2.7	6.301	0.018	0.334	9.754	<0.001	0.388
VF-Category	4.10 ± 0.91	5.14 ± 1.26	4.80 ± 0.80	4.11 ± 1.07	4.20 ± 0.83	4.00 ± 0.63	4.622	0.079	0.282	4.109	0.145	0.298
VF-Grouping	1.16 ± 1.17 ^b,c^	3.84 ± 1.58	3.21 ± 1.41	1.22 ± 1.20	1.28 ± 1.24	1.21 ± 1.11	6.482	0.032	0.196	6.278	0.038	0.214
VF-Exchange	0.60 ± 1.26 ^b,c^	1.60 ± 1.75 ^c^	1.30 ± 1.83	0.64 ± 1.44	0.80 ± 1.80	0.70 ± 1.03	8.781	0.002	0.189	8.253	0.012	0.247

Note: Values are presented as mean (M) ± standard deviation (SD); s = second; TUG = Timed Up & Go; TUGm = Timed Up & Go (manual); TUGc = Timed Up & Go (cognitive); TEC = Balance Test; LEMS = c; VF = Verbal Fluency; *p* < 0.005 = Repeated measures two-way ANOVA; ^b,c^ *p* < 0.050 = Bonferroni’s post hoc test (^b^ = considering, significant difference baseline with 24 weeks; ^c^ = considering, significant difference 12 weeks with 24 weeks); *η*^2^ = Cohen d (effect size).

## Data Availability

The data presented in this study are available upon request from the corresponding author.
